# A solid solution of ethyl and *d*_3_-methyl 2-[(4-meth­yl­pyridin-2-yl)amino]-4-(pyridin-2-yl)thia­zole-5-carboxyl­ate

**DOI:** 10.1107/S2056989020008956

**Published:** 2020-07-10

**Authors:** Andreas Beuchel, Richard Goddard, Peter Imming, Rüdiger W. Seidel

**Affiliations:** a Institut für Pharmazie, Wolfgang-Langenbeck-Str. 4, 06120 Halle (Saale), Germany; b Max-Planck-Institut für Kohlenforschung, Kaiser-Wilhelm-Platz 1, 45470 Mülheim an der Ruhr, Germany

**Keywords:** 2-amino­thia­zole, Hantzsch reaction, heterocycle, solid solution, hydrogen bonding, crystal structure

## Abstract

The synthesis of ethyl 2-[(4-methyl­pyridin-2-yl)amino)-4-(pyridin-2-yl)thia­zole- 5-carboxyl­ate *via* the Hantzsch reaction and partial *in situ* transesterification during recrystallization from methanol-*d*_4_ to the *d*_3_-methyl ester, resulting in the title solid solution, ethyl 2-[(4-methyl­pyridin-2-yl)amino)-4-(pyridin-2-yl)thia­zole-5-carboxyl­ate–*d*_3_-methyl 2-[(4-methyl­pyridin-2-yl)amino)-4-(pyridin-2-yl)thia­zole-5-carboxyl­ate (0.88/0.12), 0.88C_17_H_16_N_4_O_2_S·0.12C_16_D_3_H_11_N_4_O_2_S, is reported. The refined ratio of ethyl to *d*_3_-methyl ester in the crystal is 0.880 (6):0.120 (6). The pyridine ring is significantly twisted out of the plane of the approximately planar picoline thia­zole ester moiety. N—H⋯N hydrogen bonds between the secondary amino group and the pyridine nitro­gen atom of an adjacent symmetry-related mol­ecule link the mol­ecules into polymeric hydrogen-bonded zigzag tapes extending by glide symmetry in the [001] direction. There is structural evidence for intra­molecular N⋯S chalcogen bonding and inter­molecular weak C—H⋯O hydrogen bonds between adjacent zigzag tapes.

## Abstract 2

The crystal structure of a solid solution of ethyl and *d*_3_-methyl 2-[(4-methyl­pyridin-2-yl)amino]-4-(pyridin-2-yl)thia­zole-5-carboxyl­ate is reported.

## Chemical context   

**Figure 1:**
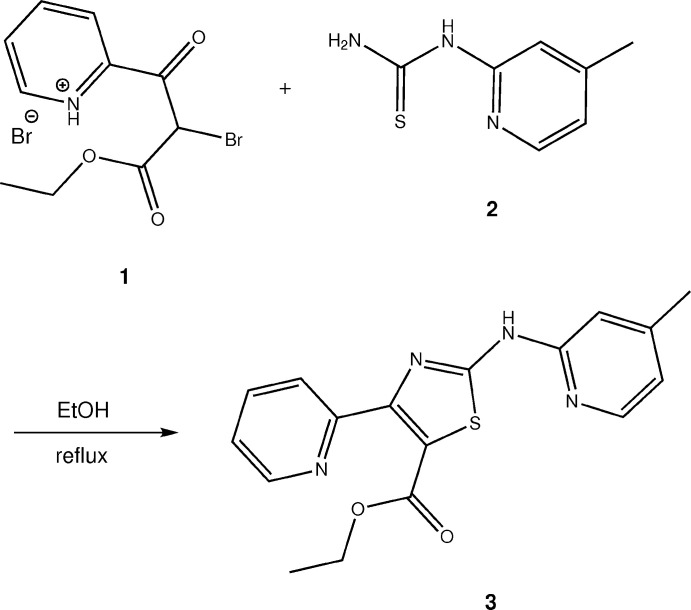
Chemical synthesis of 2-amino­thia­zole **3** from α-bromo­ketone **1** and 1-(4-methyl­pyridin-2-yl)thio­urea (**2**).

*N*,4-Diaryl-2-amino­thia­zoles were investigated based on a hit in a screening of 200,000 compounds for anti­leishmanial properties (Bhuniya *et al.*, 2015 ▸). Growth inhibition of other microorganisms by this compound class such as plasmodia (Paquet *et al.*, 2012 ▸) and mycobacteria (Kesicki *et al.*, 2016 ▸) have been reported. A 2-amino­thia­zole cluster of active compounds was discovered and formed the basis of an extensive structure–activity relationship study (Meissner *et al.*, 2013 ▸). Makam & Kannan (2014 ▸) reported a series of 2-amino­thia­zoles with a wide range of substituents at the 2-, 4- and 5-positions of the central 1,3-thia­zole ring and evaluated the inhibitory potential against *Mycobacterium tuberculosis*, H_37_Rv. Apart from desirable pharmacological effects, 2-amino­thia­zoles are also known to be cytotoxic (Meissner *et al.*, 2013 ▸). Substitution in the 5-position is a promising approach to reduce the toxicity of this compound class through hindrance of metabolic oxidation reactions in this ring position. Various synthetic routes to substituted 2-amino­thia­zoles have been described (Khalifa, 2018 ▸). The Hantzsch reaction using α-haloketones and thio­urea derivatives in polar solvents is a common method (Hantzsch & Weber, 1887 ▸; Wang, 2010 ▸). Using this method, we prepared ethyl 2-[(4-meth­yl­pyridin-2-yl)amino]-4-(pyridin-2-yl)thia­zole-5-carb­oxy­l­ate (**3**) from ethyl 2-bromo-3-oxo-3-(pyridin-2-yl)propano­ate hydro­bromide (**1**) and 1-(4-methyl­pyridin-2-yl)thio­urea (**2**) in ethanol (Fig. 1 ▸) in our ongoing optimization of compounds that inhibit the growth of *Mycobacterium abscessus*.
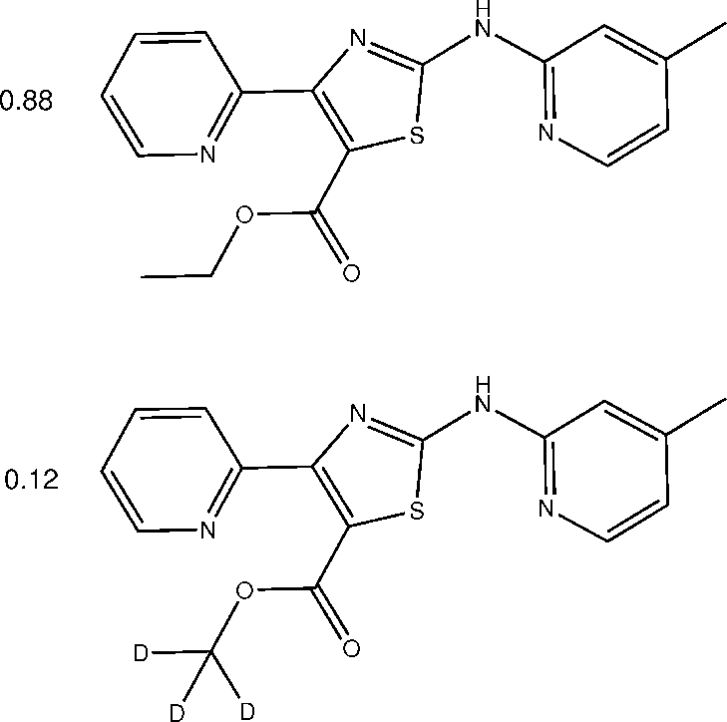


## Structural commentary   

**Figure 2:**
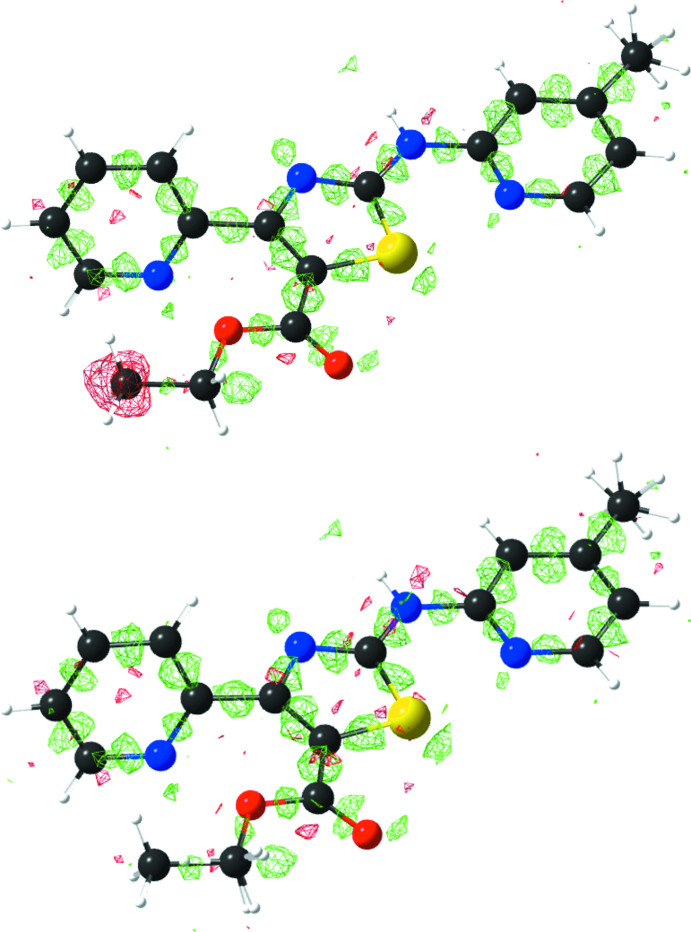
*F*_obs_–*F*_calc_ electron-density maps (isosurface level 0.18 e Å^−3^). Positive and negative residual electron density shown respectively as green and red mesh. Top: after initial structure refinement as ethyl ester **3**. Bottom: after refinement as solid solution of ethyl (**3**) and *d*_3_-methyl ester (**4**). The pictures were generated with *ShelXle* (Hübschle *et al.*, 2011 ▸).

**Figure 3:**
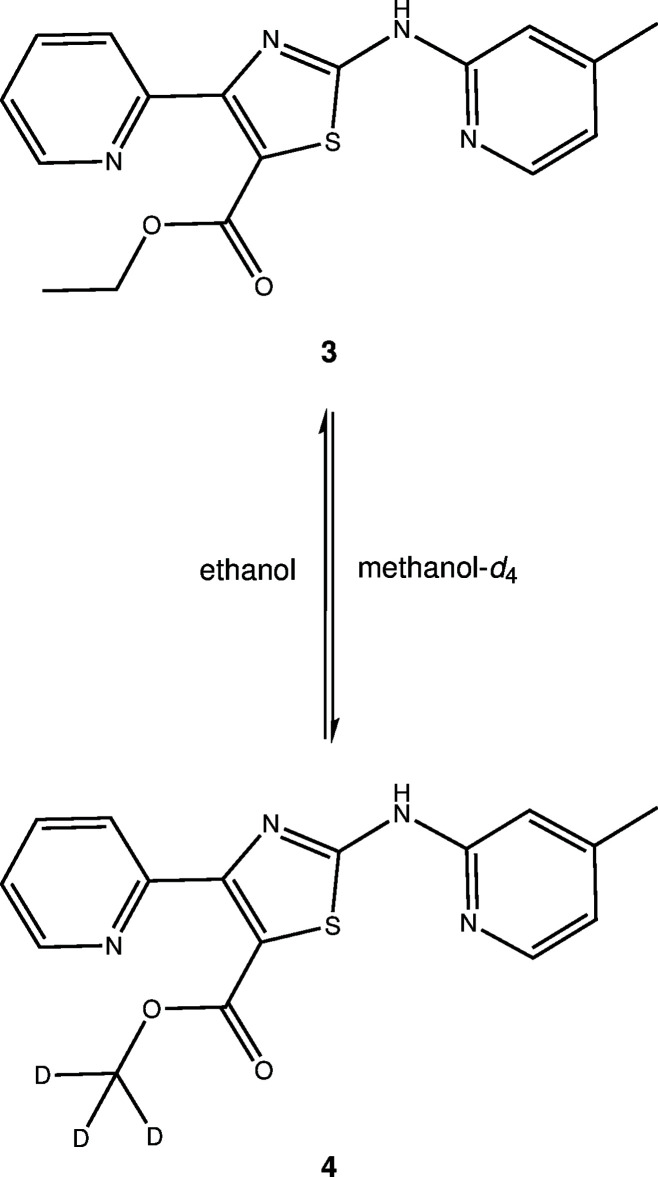
*In situ* transesterification reaction of **3** to **4** in the crystallization solvent methanol-*d*_4_.

Inspection of the difference electron-density map after initial refinement of the structure representing the anti­cipated compound **3** against the data clearly revealed unexpected negative residual electron density around C19, the methyl C atom of the ethyl ester group (Fig. 2 ▸, top), indicating that too much electron density was assigned to this site in the model. Taking the crystallization conditions (see section 5) into account, we concluded that partial *in situ* transesterification, as depicted in Fig. 3 ▸, had occurred. Methanol is known to have the strongest replacing power in transesterification reactions (Otera, 1993 ▸). After modelling the structure as a solid solution of **3** and the corresponding *d*_3_-methyl ester **4**, the negative residual electron density around C19 disappeared (Fig. 2 ▸, bottom) and the *R*_1_ factor dropped slightly from 0.0394 to 0.0383. Refinement of the occupancies yielded a ratio of 0.880 (6):0.120 (6) for **3** and **4** in the crystal. The presence of both **3** and **4** in the sample was subsequently confirmed by high-resolution mass spectrometry (see supporting information).

**Figure 4:**
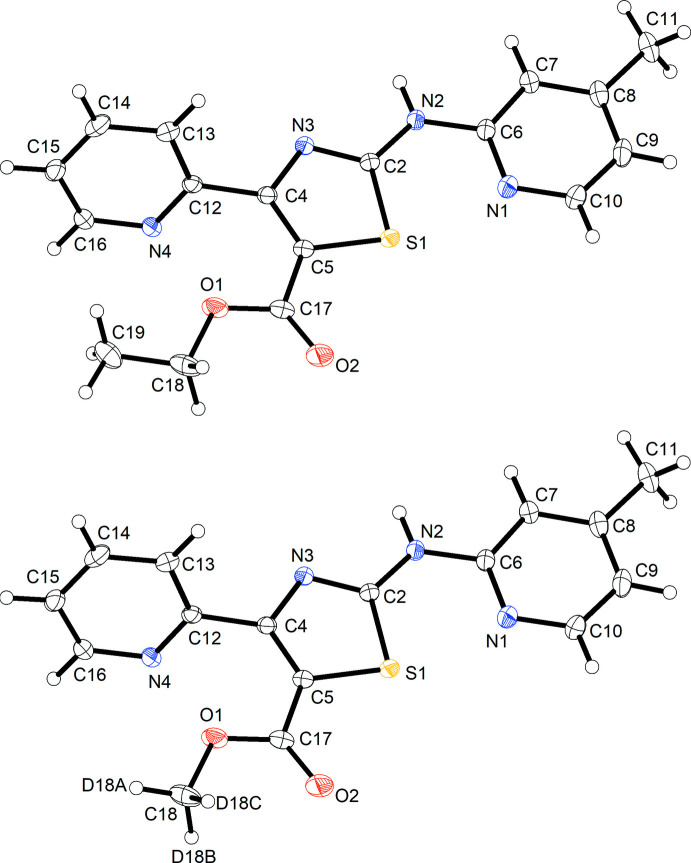
Mol­ecular structures of **3** (top) and **4** (bottom) in the crystal of the solid solution. Displacement ellipsoids are drawn at the 50% probability level. H and D atoms are represented by small spheres of arbitrary radii. Rotational disorder of the methyl group of C11 is not shown for clarity.

Fig. 4 ▸ shows the individual mol­ecular structures of **3** and **4** that make up the solid solution. Selected geometric parameters are listed in Table 1 ▸. Bond lengths and angles of the central 1,3-thia­zole five-membered heterocyclic ring are as expected (Eicher *et al.*, 2013 ▸). The thia­zole S atom and the pivot C6 atom of the picoline moiety as well as the pivot C2 atom of the thia­zole ring and the picoline nitro­gen atom N1 exhibit a synperiplanar conformation, as revealed by the respective torsion angles in Table 1 ▸. The thia­zole ring and picoline six-membered ring are nearly coplanar to one another with a dihedral angle between the respective mean planes of 3.2 (6)°. The intra­molecular S1⋯N1 distance is 2.646 (1) Å and corresponding C5—S1⋯N1 angle is 162.70 (4)°. The arrangement can structurally be regarded as a chalcogen bond between the lone pair of the picoline N atom and the σ hole at the S atom opposite to the C5—S1 bond (Scilabra *et al.*, 2019 ▸; Vogel *et al.*, 2019 ▸). The plane of the carboxyl­ate unit is tilted out of the thia­zole mean plane by 4.9 (2)°, whereas the mean plane of the pyridine ring appended to C4 is tilted out of the latter plane by 68.06 (4)°. This significant twist between the thia­zole and pyridine rings should weaken the conjugation of π electrons in the mol­ecule. Indeed, the related *N*-(4-(pyridin-3-yl)-1,3-thia­zol-2-yl)pyridin-2-amine, for example, exhibits a virtually planar mol­ecular structure in the crystal (CSD refcode: XOVJAV; Makam & Kannan, 2014 ▸). The twist between the pyridine ring and the thia­zole ring in **3** and **4** can be ascribed to involvement of the pyridine N atom in inter­molecular hydrogen bonding (see Section 3) and steric clashes with the neighbouring carboxyl­ate substituent, which appears to be preferentially conjugated to the thia­zole ring.

## Supra­molecular features   

**Figure 5:**
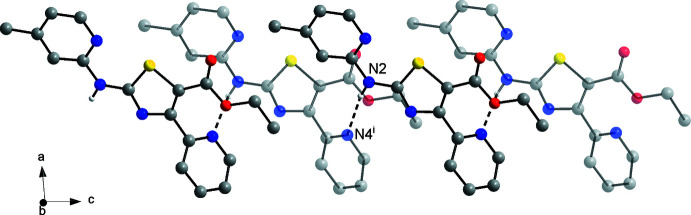
Hydrogen-bonded zigzag tape of the mol­ecules in the solid solution of **3** and **4**, shown only for the major component **3** for clarity, viewed approximately along the *b-*axis direction towards the origin. Carbon-bound H atoms are omitted for clarity. Symmetry code: (i) *x*, −*y* + 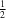, *z* − 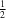.

**Figure 6:**
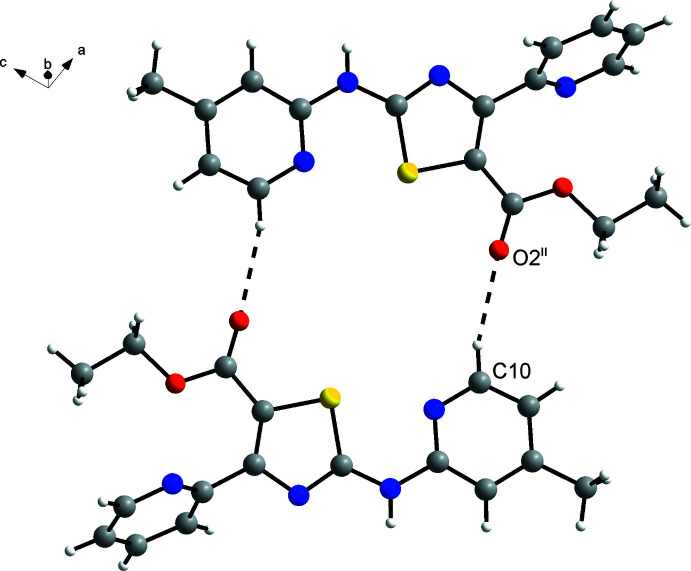
C—H⋯O hydrogen-bonded association of two adjacent mol­ecules in the solid solution of **3** and **4**, shown only for the major component **3** for clarity. For the sake of clarity, rotational disorder of the methyl groups is also not shown. Symmetry code: (ii) −*x* + 2, −*y* + 1, −*z* + 1.

The supra­molecular structure of the solid solution of **3** and **4** is dominated by hydrogen bonds of the N—H⋯N type between the secondary amino group and the pyridine N atom. As shown for the major component **3** in Fig. 5 ▸, this results in polymeric hydrogen-bonded zigzag tapes extending in the [001] direction through glide symmetry. The geometric parameters (Table 2 ▸) are within the ranges expected for strong hydrogen bonds (Thakuria *et al.*, 2017 ▸). Mol­ecules in adjacent tapes are linked through two short C—H⋯O contacts between the α-CH groups of the picoline ring and the formal C=O groups of the carboxyl­ate moieties, forming approximately planar dimeric picoline thia­zole ester units (Fig. 6 ▸). The corresponding geometric parameters (Table 2 ▸) support the inter­pretation that these are weak hydrogen bonds (Thakuria *et al.*, 2017 ▸).

## Database survey   

A search of the Cambridge Structural Database (CSD; Groom *et al.*, 2016 ▸) in June 2020 *via* WebCSD (Thomas *et al.*, 2010 ▸) revealed 15 metal-free crystal structures of 2-amino­thia­zoles with *N*-bonded heteroaromatic substituents containing a nitro­gen atom in the 2-position, all of which adopt planar mol­ecular conformations with intra­molecular N⋯S distances of 2.70 (4) Å (mean value), despite different crystal environments. These include structures of the tyrosine kinase inhib­itor dasatinib and nine of its solvates (Roy *et al.*, 2012 ▸; Sarceviča *et al.*, 2016 ▸) as well as thia­zovivin, a small-mol­ecule tool for stem-cell research (Ries *et al.*, 2013 ▸). The most related, the above-mentioned XOVJAV exhibits nearly planar N—H⋯N hydrogen-bonded dimers in the crystal structure. In contrast, in 41 crystal structures of 2-amino­thia­zoles with variously substituted *N*-phenyl groups, the two moieties are randomly orientated to one another. So far, few 5-substituted *N-*4-diaryl 2-amino­thia­zoles have been structurally characterized, *viz*. ANTZOB (Declercq *et al.*, 1981 ▸), QAWDAT (Schantl & Lagoja, 1998 ▸), VAZNEQ (Shao *et al.*, 2006 ▸), TIHKOL (Dridi & El Efrit, 2007 ▸), XIVCAJ and XIVCEN (Prevost *et al.*, 2018 ▸). As far as we are able to ascertain, there are no published crystal structures of related 5-carboxyl­ate *N-*4-diaryl 2-amino­thia­zoles, and just two for 5-carboxyl­ate *N*,*N-*4-triaryl-2-amino­thia­zoles, NIBDEJ (Souldozi *et al.*, 2013 ▸) and USAQIQ (Heydari *et al.*, 2016 ▸), in which the formal C=O group adopts an orientation anti­periplanar to the adjacent thia­zole C—S bond, in contrast to **3** and **4**.

## Synthesis and crystallization   

Syntheses of the starting materials can be found in the literature, as indicated. Solvents were of reagent grade and distilled before use. The melting point (uncorrected) was determined on a Boetius melting-point apparatus (VEB Kombinat NAGEMA, Dresden, GDR). ^1^H and ^13^C NMR spectra were recorded at room temperature on an Agilent Technologies VNMRS 400 NMR spectrometer. The residual solvent signals of DMSO-*d*6 (δ_1H_ = 2.50 ppm, δ_13C_ = 39.51 ppm) were used to reference the spectra (abbreviations: *s* = singlet, *d* = doublet, *t* = triplet, *q* = quartet, *td* = triplet of doublets, *m* = multiplet). The mass spectrum was recorded on a Q Exactive^TM^ Plus Orbitrap mass spectrometer (Thermo Scientific, Bremen, Germany), using methanol as solvent.

Compound **3** was synthesized in analogy to a procedure described by Hung *et al.* (2014 ▸): 0.18 g (0.66 mmol) of ethyl 2-bromo-3-oxo-3-(pyridin-2-yl)propano­ate hydro­bromide (**1**; Combs *et al.*, 2014 ▸) were added to a stirred solution of 0.11 g (0.66 mmol) 1-(4-methyl­pyridin-2-yl)thio­urea (**2**; Gallardo-Godoy *et al.*, 2011 ▸) in 10 mL of ethanol. The reaction mixture was heated to reflux for 16 h and then allowed to cool to room temperature. After evaporation of the solvent, the residue was taken up in 20 mL of 10% aqueous K_2_CO_3_ and extracted with 3 × 5 mL of ethyl acetate. The combined organic phases were washed with 2 × 5 mL of brine, dried over MgSO_4_, filtered and stripped of solvent under vacuum. Recrystallization from ethyl acetate yielded 43 mg (0.126 mmol, 19%) of **3**. M.p. 483 K. ^1^H NMR (400 MHz, DMSO-*d*_6_): δ 11.87 (*s*, 1H, NH), 8.59 (*m*, 1H, 6-pyridine), 8.26 (*d*, 1H, 6-picoline), 7.84 (*td*, 1H, 4-pyridine), 7.65 (*d*, 1H, 3-pyridine), 7.39 (*m*, 1H, 5-pyridine), 6.88 (*s*, 1H, 3-picoline), 6.86 (*m*, *J* = 5.3 Hz, 1H, 5-picoline), 4.11 (*q*, *J* = 7.1 Hz, 2H, CH_2_ ester), 2.29 (*s*, 3H, CH_3_ picoline), 1.12 (*t*, *J* = 7.1 Hz, 3H, CH_3_ ester) ppm. ^13^C NMR (101 MHz, DMSO-*d*_6_) δ = 162.2, 161.3, 155.5, 153.8, 151.5, 149.6, 149.1, 146.6, 136.4, 124.7, 123.8, 118.9, 115.2, 111.64, 60.72, 21.14, 14.5 ppm.

Crystals of the title solid solution of **3** and **4** suitable for X-ray analysis were obtained from a solution of **3** in methanol-*d*_4_ upon standing at room temperature for a couple of weeks. HRMS (ESI^+^): calculated for C_17_H_17_N_4_O_2_S (**3**) [*M* + H]^+^: *m*/*z* 341.10667, found: 341.10679; calculated for C_16_H_12_D_3_N_4_O_2_S (**4**) [*M* + H]^+^: *m*/*z* 330.10985, found: 330.11005 The ESI mass spectrum is shown in the supporting information.

## Refinement   

Crystal data, data collection and structure refinement details are summarized in Table 3 ▸. The ratio of the occupancies of the ethyl group belonging to **3** and the *d*_3_-methyl ester group belonging to **4** was refined by means of a free variable, resulting in 0.880 (6):0.120 (6). Carbon-bound H and D atoms were placed at geometrically calculated positions with C_aromatic_—H = 0.95 Å, C_methyl­ene_—H = 0.99 Å and C_meth­yl_—H/D = 0.98 Å and refined with *U*_iso_(H) = 1.2 *U*_eq_(C) (1.5 for methyl groups). The methyl­ene H atoms (belonging to **3**) attached to C18 were included in the split model refined for the solid solution, but the parent C18 was not. The torsion angle of the methyl group of C19 was initially determined through a circular difference-Fourier synthesis and subsequently refined while maintaining the tetra­hedral angles. The methyl group of C11 was treated as idealized disordered methyl group. Refinement of the ratio of occupancies by means of a free variable yielded 0.21 (4):0.79 (4). The amino H atom was located in a difference-Fourier map and refined semi-freely with the N—H distance restrained to a target value of 0.88 (2) Å and *U*_iso_(H) = 1.2*U*_eq_(N). The amino group was treated as non-deuterated only in agreement with the mass spectrum in methanol, although partial H/D exchange during the crystallization from methanol-*d*_4_ cannot be ruled out.

## Supplementary Material

Crystal structure: contains datablock(s) global, I. DOI: 10.1107/S2056989020008956/zl2790sup1.cif

Structure factors: contains datablock(s) 3and4. DOI: 10.1107/S2056989020008956/zl27903and4sup2.hkl

ESI mass spectrum. DOI: 10.1107/S2056989020008956/zl2790sup3.pdf

CCDC reference: 2013452

Additional supporting information:  crystallographic information; 3D view; checkCIF report

## Figures and Tables

**Table 1:** Selected geometric parameters (Å, °)

C2—N3	1.3241 (13)	C5—S1	1.7364 (11)
C2—N2	1.3653 (13)	C6—N2	1.3874 (13)
C2—S1	1.7330 (11)	O1—C17	1.3368 (15)
C4—N3	1.3678 (14)	O1—C18	1.4475 (14)
C4—C5	1.3697 (15)	C17—O2	1.2122 (15)
C4—C12	1.4852 (15)	C18—C19	1.531 (2)
			
N3—C2—N2	119.44 (10)	N3—C4—C5	115.58 (9)
N3—C2—S1	115.59 (8)	C4—C5—S1	110.42 (8)
N2—C2—S1	124.96 (8)		

**Table 2:** Hydrogen-bond geometry (Å, °)

*D*—H⋯*A*	*D*—H	H⋯*A*	*D*⋯*A*	*D*—H⋯*A*
N2—H2⋯N4^i^	0.87 (1)	2.10 (1)	2.9553 (14)	169 (1)
C10—H10⋯O2^ii^	0.95	2.47	3.3863 (16)	162

Symmetry codes: (i) 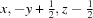; (ii) 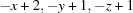.

**Table 3:** Experimental details

Crystal data	
Chemical formula	0.88C_17_H_16_N_4_O_2_S·0.12C_16_D_3_H_11_N_4_O_2_S
*M*_r_	339.08
Crystal system, space group	Monoclinic, *P*2_1_/*c*
Temperature (K)	100
*a*, *b*, *c* (Å)	9.1379 (12), 14.7534 (19), 12.1904 (16)
β (°)	94.399 (2)
*V* (Å^3^)	1638.6 (4)
*Z*	4
Radiation type	Mo *K*α
μ (mm^−1^)	0.22
Crystal size (mm)	0.09 × 0.06 × 0.02

Data collection	
Diffractometer	Bruker Kappa Mach3 APEXII
Absorption correction	Gaussian (*SADABS*; Bruker, 2012 ▸)
*T*_min_, *T*_max_	0.985, 0.997
No. of measured, independent and observed [*I* > 2σ(*I*)] reflections	44689, 5630, 4522
*R*_int_	0.051

Refinement	
*R*[*F* ^2^ > 2σ(*F* ^2^)], *wR*(*F* ^2^), *S*	0.038, 0.100, 1.04
No. of reflections	5630
No. of parameters	224
No. of restraints	1
H-atom treatment	H atoms treated by a mixture of independent and constrained refinement
Δρ_max_, Δρ_min_ (e Å^−3^)	0.46, −0.22

Computer programs: *APEX3* (Bruker, 2017 ▸) and *SAINT* (Bruker, 2004 ▸), *SHELXT2014/4* (Sheldrick, 2015*a* ▸), *SHELXL2018/3* (Sheldrick, 2015*b* ▸), *DIAMOND* (Brandenburg, 2018 ▸), *enCIFer* (Allen *et al.*, 2004 ▸) and *publCIF* (Westrip, 2010 ▸).

## supplementary crystallographic information

### Crystal data

**Table:**

0.88C_17_H_16_N_4_O_2_S·0.12C_16_D_3_H_11_N_4_O_2_S	*F*(000) = 708.2
*M**_r_* = 339.08	*D*_x_ = 1.374 Mg m^−^^3^
Monoclinic, *P*2_1_/*c*	Mo *K*α radiation, λ = 0.71073 Å
*a* = 9.1379 (12) Å	Cell parameters from 9660 reflections
*b* = 14.7534 (19) Å	θ = 2.8–31.8°
*c* = 12.1904 (16) Å	µ = 0.22 mm^−^^1^
β = 94.399 (2)°	*T* = 100 K
*V* = 1638.6 (4) Å^3^	Plate, colourless
*Z* = 4	0.09 × 0.06 × 0.02 mm

### Data collection

**Table:**

Bruker Kappa Mach3 APEXII diffractometer	5630 independent reflections
Radiation source: Incoatec IµS	4522 reflections with *I* > 2σ(*I*)
Incoatec Helios mirrors monochromator	*R*_int_ = 0.051
Detector resolution: 66.67 pixels mm^-1^	θ_max_ = 32.0°, θ_min_ = 3.0°
φ– and ω–scans	*h* = −13→13
Absorption correction: gaussian (SADABS; Bruker, 2012)	*k* = −21→21
*T*_min_ = 0.985, *T*_max_ = 0.997	*l* = −18→18
44689 measured reflections	

### Refinement

**Table:**

Refinement on *F*^2^	Primary atom site location: dual
Least-squares matrix: full	Secondary atom site location: difference Fourier map
*R*[*F*^2^ > 2σ(*F*^2^)] = 0.038	Hydrogen site location: mixed
*wR*(*F*^2^) = 0.100	H atoms treated by a mixture of independent and constrained refinement
*S* = 1.04	*w* = 1/[σ^2^(*F*_o_^2^) + (0.0439*P*)^2^ + 0.6165*P*] where *P* = (*F*_o_^2^ + 2*F*_c_^2^)/3
5630 reflections	(Δ/σ)_max_ = 0.001
224 parameters	Δρ_max_ = 0.46 e Å^−^^3^
1 restraint	Δρ_min_ = −0.22 e Å^−^^3^

### Special details

**Table:**

Geometry. All esds (except the esd in the dihedral angle between two l.s. planes) are estimated using the full covariance matrix. The cell esds are taken into account individually in the estimation of esds in distances, angles and torsion angles; correlations between esds in cell parameters are only used when they are defined by crystal symmetry. An approximate (isotropic) treatment of cell esds is used for estimating esds involving l.s. planes.

### Fractional atomic coordinates and isotropic or equivalent isotropic displacement parameters (Å^2^)

**Table:**

	*x*	*y*	*z*	*U*_iso_*/*U*_eq_	Occ. (<1)
C2	0.59057 (12)	0.34576 (7)	0.35409 (9)	0.01513 (19)	
C4	0.46550 (11)	0.34523 (7)	0.50197 (9)	0.01489 (19)	
C5	0.58680 (12)	0.39179 (7)	0.54400 (9)	0.01575 (19)	
C6	0.73603 (12)	0.35354 (8)	0.19499 (9)	0.0167 (2)	
C7	0.74804 (12)	0.33176 (8)	0.08384 (9)	0.0180 (2)	
H7	0.673642	0.297799	0.043611	0.022*	
C8	0.87112 (13)	0.36105 (8)	0.03440 (10)	0.0205 (2)	
C9	0.97523 (14)	0.41284 (9)	0.09736 (11)	0.0245 (2)	
H9	1.059591	0.435434	0.065460	0.029*	
C10	0.95399 (13)	0.43066 (9)	0.20600 (11)	0.0237 (2)	
H10	1.025698	0.465559	0.247758	0.028*	
C11	0.89674 (15)	0.33555 (9)	−0.08237 (10)	0.0265 (3)	
H11A	0.994097	0.356530	−0.099652	0.040*	0.213 (18)
H11B	0.821645	0.364006	−0.132801	0.040*	0.213 (18)
H11C	0.891132	0.269531	−0.090548	0.040*	0.213 (18)
H11D	0.810486	0.303514	−0.115682	0.040*	0.787 (18)
H11E	0.982938	0.296039	−0.082533	0.040*	0.787 (18)
H11F	0.913451	0.390514	−0.124786	0.040*	0.787 (18)
C12	0.33298 (12)	0.32273 (8)	0.55988 (8)	0.0156 (2)	
C13	0.20035 (12)	0.36487 (8)	0.52774 (10)	0.0193 (2)	
H13	0.191964	0.404257	0.465922	0.023*	
C14	0.08061 (13)	0.34797 (9)	0.58819 (11)	0.0236 (2)	
H14	−0.010565	0.377382	0.570229	0.028*	
C15	0.09633 (13)	0.28763 (9)	0.67492 (10)	0.0237 (2)	
H15	0.016194	0.275033	0.717791	0.028*	
C16	0.23115 (13)	0.24566 (10)	0.69849 (10)	0.0244 (3)	
H16	0.240167	0.202767	0.756738	0.029*	
O1	0.51067 (10)	0.42378 (7)	0.71754 (7)	0.02527 (19)	0.880 (6)
C17	0.62170 (13)	0.43211 (8)	0.65294 (9)	0.0190 (2)	0.880 (6)
C18	0.53174 (16)	0.45723 (10)	0.82926 (10)	0.0290 (3)	0.880 (6)
H18A	0.536392	0.524271	0.829953	0.035*	0.880 (6)
H18B	0.623956	0.433108	0.866076	0.035*	0.880 (6)
C19	0.39913 (19)	0.42398 (11)	0.88747 (12)	0.0289 (4)	0.880 (6)
H19A	0.308801	0.447409	0.849093	0.043*	0.880 (6)
H19B	0.406760	0.445679	0.963673	0.043*	0.880 (6)
H19C	0.396954	0.357569	0.886890	0.043*	0.880 (6)
O1'	0.51067 (10)	0.42378 (7)	0.71754 (7)	0.02527 (19)	0.120 (6)
C17'	0.62170 (13)	0.43211 (8)	0.65294 (9)	0.0190 (2)	0.120 (6)
C18'	0.53174 (16)	0.45723 (10)	0.82926 (10)	0.0290 (3)	0.120 (6)
D18A	0.442345	0.446863	0.867031	0.044*	0.120 (6)
D18B	0.614053	0.425167	0.868235	0.044*	0.120 (6)
D18C	0.553091	0.522305	0.828159	0.044*	0.120 (6)
S1	0.71130 (3)	0.40479 (2)	0.44447 (2)	0.01604 (7)	
N1	0.83635 (11)	0.40094 (7)	0.25585 (8)	0.02034 (19)	
N2	0.61495 (10)	0.32512 (7)	0.24785 (8)	0.01741 (18)	
H2	0.5448 (15)	0.2970 (10)	0.2099 (12)	0.021*	
N3	0.46710 (10)	0.31919 (7)	0.39439 (7)	0.01658 (18)	
N4	0.34941 (10)	0.26282 (7)	0.64283 (8)	0.0204 (2)	
O2	0.73781 (11)	0.46826 (7)	0.68069 (8)	0.0327 (2)	

### Atomic displacement parameters (Å^2^)

**Table:**

	*U*^11^	*U*^22^	*U*^33^	*U*^12^	*U*^13^	*U*^23^
C2	0.0145 (4)	0.0163 (5)	0.0143 (4)	−0.0014 (4)	−0.0002 (4)	−0.0009 (4)
C4	0.0143 (4)	0.0169 (5)	0.0133 (4)	0.0013 (4)	−0.0004 (3)	−0.0002 (4)
C5	0.0161 (5)	0.0170 (5)	0.0138 (4)	0.0002 (4)	−0.0010 (4)	−0.0004 (4)
C6	0.0156 (5)	0.0171 (5)	0.0177 (5)	−0.0009 (4)	0.0033 (4)	0.0013 (4)
C7	0.0189 (5)	0.0181 (5)	0.0172 (5)	−0.0007 (4)	0.0040 (4)	0.0005 (4)
C8	0.0232 (5)	0.0176 (5)	0.0216 (5)	0.0015 (4)	0.0083 (4)	0.0037 (4)
C9	0.0212 (5)	0.0245 (6)	0.0288 (6)	−0.0028 (5)	0.0090 (5)	0.0054 (5)
C10	0.0188 (5)	0.0257 (6)	0.0267 (6)	−0.0062 (4)	0.0029 (4)	0.0020 (5)
C11	0.0333 (7)	0.0258 (6)	0.0220 (6)	0.0011 (5)	0.0132 (5)	0.0024 (5)
C12	0.0145 (4)	0.0195 (5)	0.0126 (4)	−0.0001 (4)	−0.0002 (3)	−0.0032 (4)
C13	0.0164 (5)	0.0204 (5)	0.0206 (5)	−0.0001 (4)	−0.0024 (4)	−0.0006 (4)
C14	0.0135 (5)	0.0274 (6)	0.0294 (6)	0.0015 (4)	−0.0011 (4)	−0.0049 (5)
C15	0.0151 (5)	0.0375 (7)	0.0188 (5)	−0.0027 (5)	0.0033 (4)	−0.0052 (5)
C16	0.0178 (5)	0.0406 (7)	0.0150 (5)	−0.0010 (5)	0.0016 (4)	0.0049 (5)
O1	0.0238 (4)	0.0370 (5)	0.0146 (4)	0.0024 (4)	−0.0008 (3)	−0.0088 (3)
C17	0.0231 (5)	0.0182 (5)	0.0151 (5)	0.0010 (4)	−0.0038 (4)	−0.0011 (4)
C18	0.0384 (7)	0.0324 (7)	0.0153 (5)	0.0078 (6)	−0.0040 (5)	−0.0080 (5)
C19	0.0403 (9)	0.0292 (8)	0.0178 (7)	0.0047 (6)	0.0062 (6)	−0.0039 (5)
O1'	0.0238 (4)	0.0370 (5)	0.0146 (4)	0.0024 (4)	−0.0008 (3)	−0.0088 (3)
C17'	0.0231 (5)	0.0182 (5)	0.0151 (5)	0.0010 (4)	−0.0038 (4)	−0.0011 (4)
C18'	0.0384 (7)	0.0324 (7)	0.0153 (5)	0.0078 (6)	−0.0040 (5)	−0.0080 (5)
S1	0.01480 (12)	0.01736 (13)	0.01559 (12)	−0.00301 (9)	−0.00120 (9)	−0.00039 (9)
N1	0.0182 (4)	0.0222 (5)	0.0207 (5)	−0.0045 (4)	0.0023 (4)	0.0004 (4)
N2	0.0158 (4)	0.0225 (5)	0.0143 (4)	−0.0051 (4)	0.0028 (3)	−0.0026 (3)
N3	0.0147 (4)	0.0220 (5)	0.0130 (4)	−0.0026 (3)	0.0011 (3)	−0.0018 (3)
N4	0.0153 (4)	0.0313 (5)	0.0146 (4)	0.0019 (4)	0.0014 (3)	0.0038 (4)
O2	0.0337 (5)	0.0416 (6)	0.0217 (4)	−0.0163 (4)	−0.0050 (4)	−0.0051 (4)

### Geometric parameters (Å, º)

**Table:**

C2—N3	1.3241 (13)	C12—N4	1.3427 (14)
C2—N2	1.3653 (13)	C12—C13	1.3914 (15)
C2—S1	1.7330 (11)	C13—C14	1.3879 (16)
C4—N3	1.3678 (14)	C13—H13	0.9500
C4—C5	1.3697 (15)	C14—C15	1.3812 (19)
C4—C12	1.4852 (15)	C14—H14	0.9500
C5—C17'	1.4677 (15)	C15—C16	1.3891 (17)
C5—C17	1.4677 (15)	C15—H15	0.9500
C5—S1	1.7364 (11)	C16—N4	1.3434 (14)
C6—N1	1.3324 (15)	C16—H16	0.9500
C6—N2	1.3874 (13)	O1—C17	1.3368 (15)
C6—C7	1.4050 (15)	O1—C18	1.4475 (14)
C7—C8	1.3852 (15)	C17—O2	1.2122 (15)
C7—H7	0.9500	C18—C19	1.531 (2)
C8—C9	1.4021 (18)	C18—H18A	0.9900
C8—C11	1.5080 (16)	C18—H18B	0.9900
C9—C10	1.3785 (18)	C19—H19A	0.9800
C9—H9	0.9500	C19—H19B	0.9800
C10—N1	1.3481 (15)	C19—H19C	0.9800
C10—H10	0.9500	O1'—C17'	1.3368 (15)
C11—H11A	0.9800	O1'—C18'	1.4475 (14)
C11—H11B	0.9800	C17'—O2	1.2122 (15)
C11—H11C	0.9800	C18'—D18A	0.9800
C11—H11D	0.9800	C18'—D18B	0.9800
C11—H11E	0.9800	C18'—D18C	0.9800
C11—H11F	0.9800	N2—H2	0.867 (12)
			
N3—C2—N2	119.44 (10)	N4—C12—C4	117.19 (9)
N3—C2—S1	115.59 (8)	C13—C12—C4	119.38 (10)
N2—C2—S1	124.96 (8)	C14—C13—C12	118.38 (11)
N3—C4—C5	115.58 (9)	C14—C13—H13	120.8
N3—C4—C12	117.52 (9)	C12—C13—H13	120.8
C5—C4—C12	126.88 (10)	C15—C14—C13	118.84 (11)
C4—C5—C17'	130.93 (10)	C15—C14—H14	120.6
C4—C5—C17	130.93 (10)	C13—C14—H14	120.6
C4—C5—S1	110.42 (8)	C14—C15—C16	118.95 (11)
C17'—C5—S1	118.62 (8)	C14—C15—H15	120.5
C17—C5—S1	118.62 (8)	C16—C15—H15	120.5
N1—C6—N2	116.05 (10)	N4—C16—C15	123.13 (12)
N1—C6—C7	123.77 (10)	N4—C16—H16	118.4
N2—C6—C7	120.19 (10)	C15—C16—H16	118.4
C8—C7—C6	118.31 (11)	C17—O1—C18	118.08 (10)
C8—C7—H7	120.8	O2—C17—O1	124.31 (11)
C6—C7—H7	120.8	O2—C17—C5	123.75 (11)
C7—C8—C9	118.12 (11)	O1—C17—C5	111.94 (10)
C7—C8—C11	121.52 (11)	O1—C18—C19	105.90 (11)
C9—C8—C11	120.31 (11)	O1—C18—H18A	110.6
C10—C9—C8	119.35 (11)	C19—C18—H18A	110.6
C10—C9—H9	120.3	O1—C18—H18B	110.6
C8—C9—H9	120.3	C19—C18—H18B	110.6
N1—C10—C9	123.16 (12)	H18A—C18—H18B	108.7
N1—C10—H10	118.4	C18—C19—H19A	109.5
C9—C10—H10	118.4	C18—C19—H19B	109.5
C8—C11—H11A	109.5	H19A—C19—H19B	109.5
C8—C11—H11B	109.5	C18—C19—H19C	109.5
H11A—C11—H11B	109.5	H19A—C19—H19C	109.5
C8—C11—H11C	109.5	H19B—C19—H19C	109.5
H11A—C11—H11C	109.5	C17'—O1'—C18'	118.08 (10)
H11B—C11—H11C	109.5	O2—C17'—O1'	124.31 (11)
C8—C11—H11D	109.5	O2—C17'—C5	123.75 (11)
H11A—C11—H11D	141.1	O1'—C17'—C5	111.94 (10)
H11B—C11—H11D	56.3	O1'—C18'—D18A	109.5
H11C—C11—H11D	56.3	O1'—C18'—D18B	109.5
C8—C11—H11E	109.5	D18A—C18'—D18B	109.5
H11A—C11—H11E	56.3	O1'—C18'—D18C	109.5
H11B—C11—H11E	141.1	D18A—C18'—D18C	109.5
H11C—C11—H11E	56.3	D18B—C18'—D18C	109.5
H11D—C11—H11E	109.5	C2—S1—C5	88.25 (5)
C8—C11—H11F	109.5	C6—N1—C10	117.26 (10)
H11A—C11—H11F	56.3	C2—N2—C6	124.55 (10)
H11B—C11—H11F	56.3	C2—N2—H2	116.4 (10)
H11C—C11—H11F	141.1	C6—N2—H2	118.7 (10)
H11D—C11—H11F	109.5	C2—N3—C4	110.16 (9)
H11E—C11—H11F	109.5	C12—N4—C16	117.17 (10)
N4—C12—C13	123.42 (10)		
			
N3—C4—C5—C17'	178.00 (11)	S1—C5—C17—O1	174.38 (8)
C12—C4—C5—C17'	−0.3 (2)	C17—O1—C18—C19	−170.48 (11)
N3—C4—C5—C17	178.00 (11)	C18'—O1'—C17'—O2	−2.22 (18)
C12—C4—C5—C17	−0.3 (2)	C18'—O1'—C17'—C5	177.51 (10)
N3—C4—C5—S1	0.17 (13)	C4—C5—C17'—O2	176.43 (13)
C12—C4—C5—S1	−178.13 (9)	S1—C5—C17'—O2	−5.89 (16)
N1—C6—C7—C8	0.05 (18)	C4—C5—C17'—O1'	−3.30 (18)
N2—C6—C7—C8	−179.65 (10)	S1—C5—C17'—O1'	174.38 (8)
C6—C7—C8—C9	−1.58 (17)	N3—C2—S1—C5	0.50 (9)
C6—C7—C8—C11	176.13 (11)	N2—C2—S1—C5	−178.93 (10)
C7—C8—C9—C10	1.73 (18)	C4—C5—S1—C2	−0.36 (9)
C11—C8—C9—C10	−176.02 (12)	C17'—C5—S1—C2	−178.49 (9)
C8—C9—C10—N1	−0.3 (2)	C17—C5—S1—C2	−178.49 (9)
N3—C4—C12—N4	113.71 (12)	N2—C6—N1—C10	−178.95 (11)
C5—C4—C12—N4	−68.02 (15)	C7—C6—N1—C10	1.34 (18)
N3—C4—C12—C13	−67.32 (14)	C9—C10—N1—C6	−1.20 (19)
C5—C4—C12—C13	110.95 (13)	N3—C2—N2—C6	176.27 (10)
N4—C12—C13—C14	3.63 (17)	S1—C2—N2—C6	−4.31 (17)
C4—C12—C13—C14	−175.28 (10)	N1—C6—N2—C2	2.92 (17)
C12—C13—C14—C15	−2.39 (18)	C7—C6—N2—C2	−177.36 (11)
C13—C14—C15—C16	−0.24 (19)	N2—C2—N3—C4	178.98 (10)
C14—C15—C16—N4	2.0 (2)	S1—C2—N3—C4	−0.49 (13)
C18—O1—C17—O2	−2.22 (18)	C5—C4—N3—C2	0.20 (14)
C18—O1—C17—C5	177.51 (10)	C12—C4—N3—C2	178.66 (10)
C4—C5—C17—O2	176.43 (13)	C13—C12—N4—C16	−1.93 (17)
S1—C5—C17—O2	−5.89 (16)	C4—C12—N4—C16	176.99 (11)
C4—C5—C17—O1	−3.30 (18)	C15—C16—N4—C12	−0.95 (19)

### Hydrogen-bond geometry (Å, º)

**Table:**

*D*—H···*A*	*D*—H	H···*A*	*D*···*A*	*D*—H···*A*
N2—H2···N4^i^	0.87 (1)	2.10 (1)	2.9553 (14)	169 (1)
C10—H10···O2^ii^	0.95	2.47	3.3863 (16)	162

Symmetry codes: (i) *x*, −*y*+1/2, *z*−1/2; (ii) −*x*+2, −*y*+1, −*z*+1.
