# Supplementary material for: A solid solution of ethyl and d 3-methyl 2-[(4-meth­yl­pyridin-2-yl)amino]-4-(pyridin-2-yl)thia­zole-5-carboxyl­ate
Source: Acta Crystallogr E Crystallogr Commun. 2020 Jul 10;76(Pt 8):1255–9. doi: 10.1107/S2056989020008956 (PMC7405567; doi:10.1107/S2056989020008956)
Supplement: Supplementary file 3 [file e-76-01255-sup3.pdf]

electrospray-ionization (Sol.: CH3OH) pos. ions

molecular weight 329, 340

characteristical ions
330 = [329 + H]
341 = [340 + H]
352 = [329 + Na]
363 = [340 + Na]

3.09.2018
File: 139204a-00.RAW

Analyse: EMC-EA-615-01
LMN: Chemische Kristallographie

Ionisierung: ESIpos
Lösungsmittel:CH3OH
Spektrometer: Exactive

Auswerter: Kampen (2243)

Suggestions:

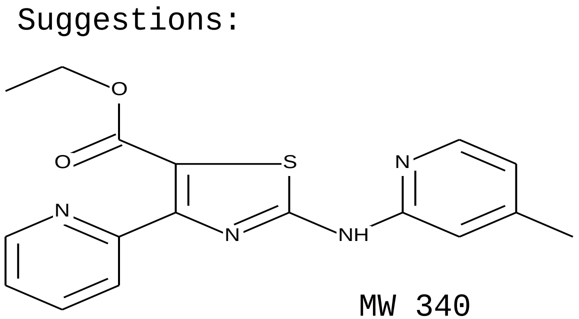

MW 340

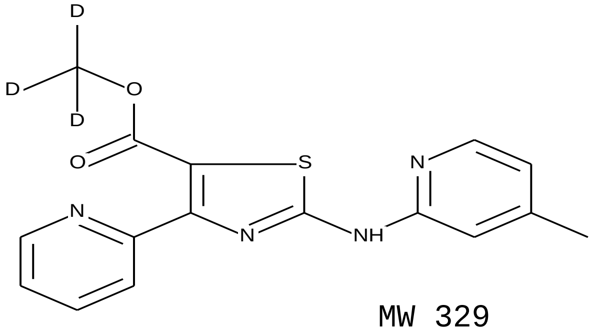

MW 329

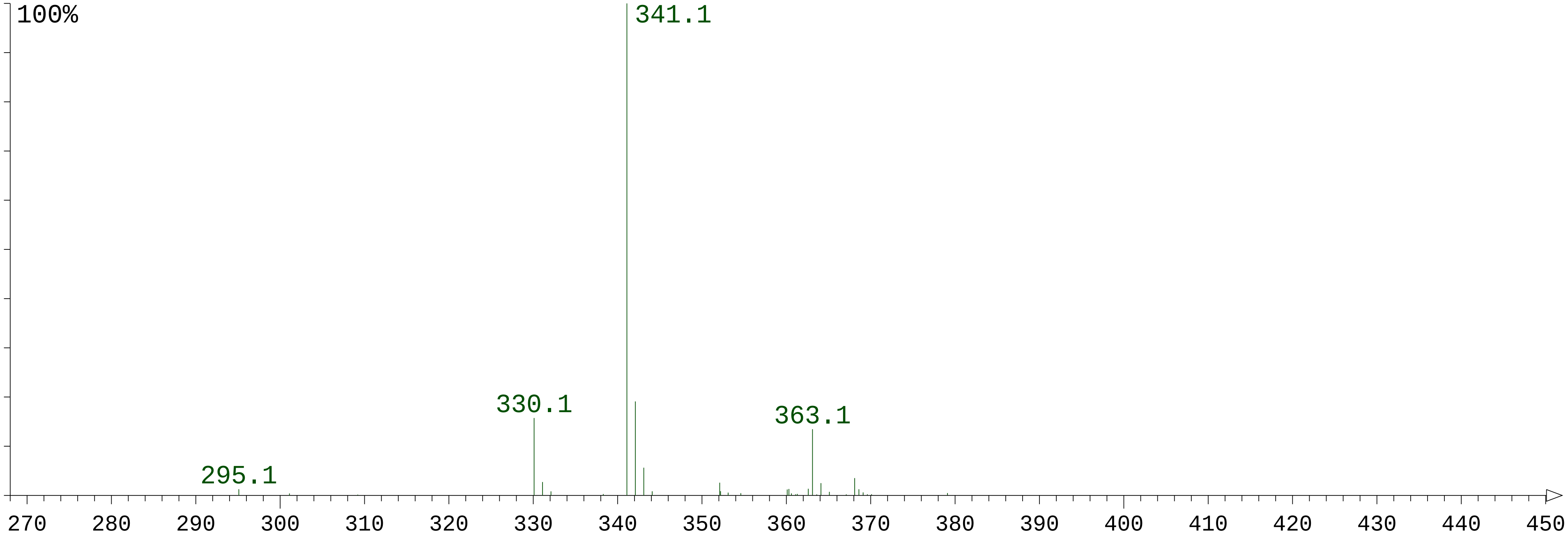

R14: Edit:R3: 56(1:55)(o:83) m(55-66) 34403 49% 139204a-00 * EMC-EA-615-01/ESIpos+neg/CH2Cl

Mass to be matched (m/z): 341.106790 Charge: 1

Mass Tolerance: ±0.005000
Restriction of atom numbers:

C	H	N	O	S
1-100	1-100	4-4	max 10	1-1

Number of calculated Formulas: 1

Formula	Diff.(ppm)	theor. m/z
C17 H17 N4 O2 S1	-0.35	341.106672

Datum	3.09.2018
Analyse:	139204b-00
Sigel:	EMC-EA-615-01
LMN: Chemische Kristallographie	
Messung:	Massenfeinbestimmung
Methode:	ESIpos
Lösungsmittel:	CH3OH
Spektrometer:	Exactive
Auswerter:	Kampen (2243)

Suggestion:
C17H16N4O2S1 MW 340

characteristical ion
341 = [340 + H]

Mass to be matched (m/z): 330.110050 Charge: 1

Mass Tolerance: ±0.005000
Restriction of atom numbers:

C	H	D	N	O	S
1-100	1-100	max 3	4-4	max 10	1-1

Number of calculated Formulas: 4

Formula	Diff.(ppm)	theor. m/z
C16 H12 D3 N4 O2 S1	-0.60	330.109854
C16 H14 D2 N4 O2 S1	4.09	330.111401
C16 H16 D1 N4 O2 S1	8.78	330.112949
C16 H18 N4 O2 S1	13.47	330.114497

Datum	3.09.2018
Analyse:	139204c-00
Sigel:	EMC-EA-615-01
LMN: Chemische Kristallographie	
Messung:	Massenfeinbestimmung
Methode:	ESIpos
Lösungsmittel:	CH3OH
Spektrometer:	Exactive
Auswerter:	Kampen (2243)

Suggestion:
C16H11D3N4O2S1 MW 329

characteristical ion
330 = [329 + H]
